# *catena*-Poly[[[aqua­copper(II)]-μ-(biphenyl-2,2′-di­carboxyl­ato)-μ-[*N*,*N*′-bis­(pyridin-4-yl)urea]] 1.25-hydrate]

**DOI:** 10.1107/S2414314620005891

**Published:** 2020-05-05

**Authors:** Ben Uzorka, Robert L. LaDuca

**Affiliations:** a E-35 Holmes Hall, Michigan State University, Lyman Briggs College, 919 E. Shaw Lane, East Lansing, MI 48825, USA; University of Toronto, Canada

**Keywords:** crystal structure

## Abstract

In the title compound, {[Cu(C_14_H_8_O_4_)(C_11_H_10_N_4_O)(H_2_O)]·1.25H_2_O}_*n*_, the Cu^II^ cations are coordinated in a square-pyramidal fashion by *trans* carboxyl­ate O-atom donors from two diphenate (dip) ligands, *trans* pyridyl N-atom donors from two bis­(4-pyrid­yl)urea (bpu) ligands, and a ligated water mol­ecule in the apical position. [Cu(H_2_O)(dip)(bpu)]_*n*_ coordination polymer layer motifs are oriented parallel to (  02). These layer motifs display a standard (4,4) rectangular grid topology and stack in an *AAA* pattern along the *a*-axis direction to form the full three-dimensional crystal structure of the title compound, mediated by N—H⋯O and O—H⋯O hydrogen bonding patterns involving the water mol­ecules of crystallization.

## Abstract 2

A divalent copper two-dimensional coordination polymer, {[Cu(H_2_O)(diphenate)(bis­(4-pyrid­yl)urea)]·1.25H_2_O}_*n*_, was structurally characterized by single-crystal X-ray diffraction.

## Graphical abstract

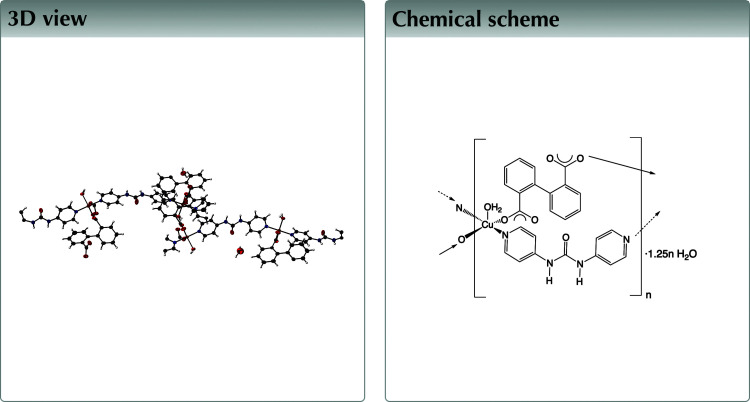

## Structure description

The title compound was isolated during an exploratory synthetic effort aiming to produce a copper coordination polymer containing both diphenate (dip) and bis­(4-pyrid­yl)urea (bpu) ligands. The bpu ligand has seldom been used in coordination polymer chemistry to date (Kumar *et al.*, 2007 ▸). Our group recently published a series of zinc diphenate coordination polymers that acted as turn-off luminescence sensors for nitroaromatic detection analyses (Martinez, Shrode, *et al.*, 2018 ▸)

**Figure 1:**
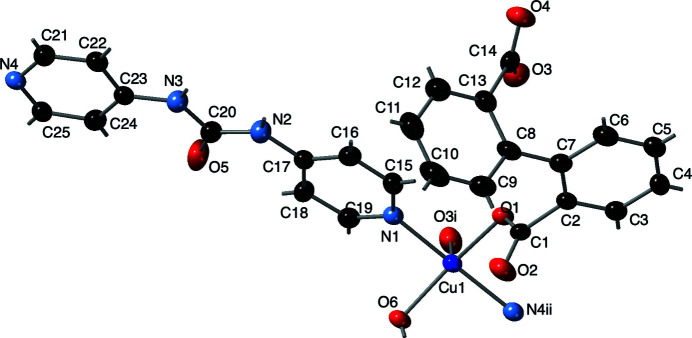
The coordination environment of the title compound, showing the distorted square-pyramidal coordination at the Cu1 atom. Displacement ellipsoids are drawn at the 50% probability level. Color code: Cu, dark blue; O, red; N, light blue; C, black. H atom positions are shown as sticks [symmetry code: (ii) *x* + 1, −*y* + 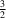 , *z* + 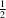 ].

**Figure 2:**
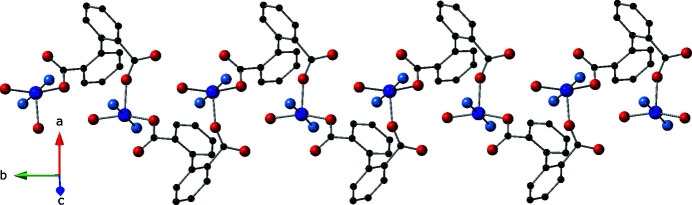
The [Cu(H_2_O)(dip)]_*n*_ chain motif in the title compound, oriented parallel to *b*.

**Figure 3:**
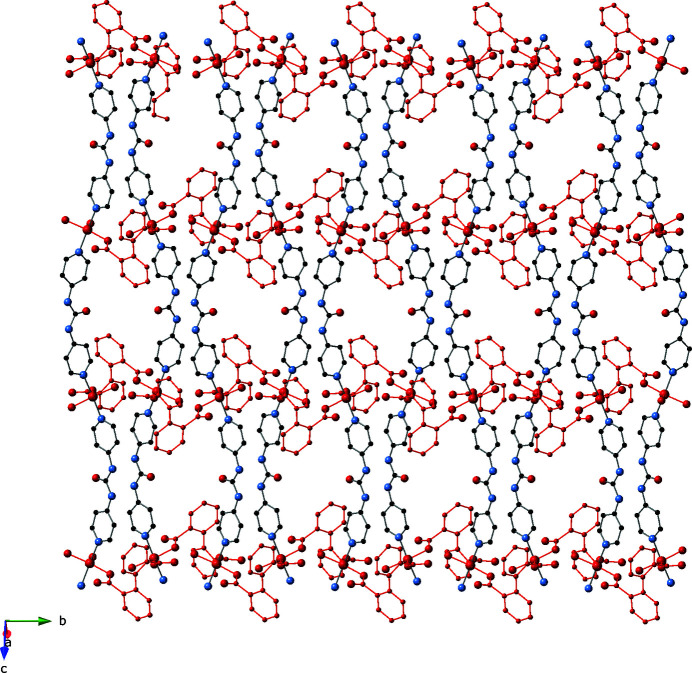
A face-view perspective of the two-dimensional [Cu(H_2_O)(dip)(bpu)]_*n*_ coordination polymer layer motif in the title compound. [Cu(H_2_O)(dip)]_*n*_ chain motifs are drawn in red.

**Figure 4:**
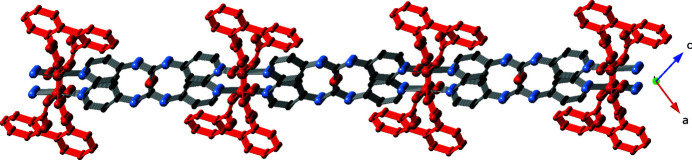
A side-view perspective of the two-dimensional [Cu(H_2_O)(dip)(bpu)]_*n*_ coordination polymer layer motif in the title compound. [Cu(H_2_O)(dip)]_*n*_ chain motifs are drawn in red.

**Figure 5:**
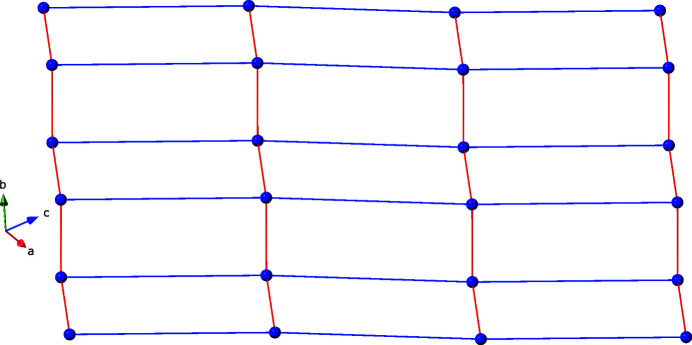
Schematic perspective of the (4,4) rectangular grid topology in the title compound. The spheres represent the copper atoms, the red lines represent the dip ligands, and the blue lines represent the bpu ligands.

The asymmetric unit of the title compound contains a Cu^II^ ion, a bound water mol­ecule, a fully deprotonated diphenate (dip) ligand, a bis­(4-pyrid­yl)urea (bpu) ligand, a water mol­ecule of crystallization best refined at full occupancy, and a water mol­ecule of crystallization best refined at one-quarter occupancy. The Cu^II^ ion displays a {CuN_2_O_3_} square-pyramidal coordination environment (Fig. 1 ▸), with the bound water mol­ecule in the elongated apical position. The basal plane is defined by *trans* carboxyl­ate O-atom donors from two dip ligands, and *trans* pyridyl N-atom donors from two bpu ligands. Bond lengths and angles within the coordination environment are listed in Table 1 ▸. Adjacent [Cu(H_2_O)]^2+^ coordination fragments are connected by dip ligands into [Cu(H_2_O)(dip)]_*n*_ chain motifs, which are oriented parallel to *b* (Fig. 2 ▸). The Cu⋯Cu inter­nuclear distance within the chain motifs measures 5.394 (1) Å. The dip ligands show an inter-ring twist of 112.7°. In turn, the [Cu(H_2_O)(dip)]_*n*_ chain motifs are pillared by dipodal bpu ligands into two-dimensional [Cu(H_2_O)(dip)(bpu)]_*n*_ coordination polymer layer motifs that are situated parallel to the [ 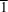 02] crystal planes (Fig. 3 ▸). The Cu⋯Cu inter­nuclear distance spanned by the bpu ligands measures 14.03 (1) Å. A side view of a single layer motif is shown in Fig. 4 ▸. If the copper atoms are considered to be 4-connected nodes, and the organic ligands considered as simple linkers, the topology of the layer is that of a common (4,4) rectangular grid (Fig. 5 ▸).

**Figure 6:**
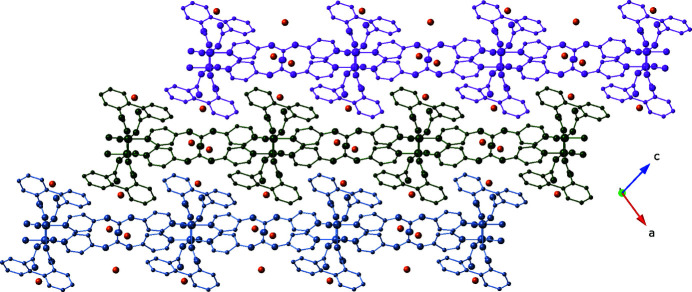
*AAA* stacking of [Cu(dip)(bpu)]_*n*_ coordination polymer layer motifs in the title compound.

Adjacent [Cu(H_2_O)(dip)(bpu)]_n_ layers stack in an *AAA* pattern along the *a*-axis direction (Fig. 6 ▸). Inter­layer aggregation is caused by hydrogen-bonding pathways involving the water mol­ecules of crystallization (O1*W*). N—H⋯O hydrogen-bonding donation between bpu N—H groups and water mol­ecules of crystallization anchors these water mol­ecules to one coordination polymer layer. In turn, the water mol­ecules of crystallization donate O—H⋯O hydrogen bonds to unligated dip carboxyl­ate O atoms in the neighboring layer. Details regarding the hydrogen bonding in the title compound are listed in Table 2 ▸.

## Synthesis and crystallization

Cu(NO_3_)_2_·2.5 H_2_O (87 mg, 0.37 mmol), diphenic acid (90 mg, 0.37 mmol), bis­(4-pyrid­yl)urea (79 mg, 0.37 mmol) and 0.75 ml of a 1.0 *M* NaOH solution were placed into 10 ml of distilled H_2_O in a Teflon-lined acid digestion bomb. The bomb was sealed and heated in an oven at 373 K for 24 h, and then cooled slowly to 273 K. Green–blue crystals of the title complex were obtained along with a flocculent green powder.

## Refinement

Crystal data, data collection and structure refinement details are summarized in Table 3 ▸.

## Supplementary Material

Crystal structure: contains datablock(s) I. DOI: 10.1107/S2414314620005891/lh4053sup1.cif

Structure factors: contains datablock(s) I. DOI: 10.1107/S2414314620005891/lh4053Isup2.hkl

CCDC reference: 1999774

Additional supporting information:  crystallographic information; 3D view; checkCIF report

## Figures and Tables

**Table 1:** Selected geometric parameters (Å, °)

Cu1—O1	1.960 (2)	Cu1—N1	2.003 (3)
Cu1—O3^i^	2.205 (3)	Cu1—N4^ii^	2.016 (3)
Cu1—O6	1.981 (2)		
			
O1—Cu1—O3^i^	105.34 (10)	O6—Cu1—N1	88.00 (11)
O1—Cu1—O6	162.28 (11)	O6—Cu1—N4^ii^	93.20 (11)
O1—Cu1—N1	91.02 (11)	N1—Cu1—O3^i^	93.24 (12)
O1—Cu1—N4^ii^	86.57 (11)	N1—Cu1—N4^ii^	175.68 (12)
O6—Cu1—O3^i^	92.38 (10)	N4^ii^—Cu1—O3^i^	90.86 (12)

Symmetry codes: (i) 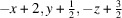 ; (ii) 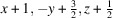 .

**Table 2:** Hydrogen-bond geometry (Å, °)

*D*—H⋯*A*	*D*—H	H⋯*A*	*D*⋯*A*	*D*—H⋯*A*
O6—H6*A*⋯O1^i^	0.89	1.84	2.650 (3)	152
O6—H6*B*⋯O4^iii^	0.89	1.78	2.598 (4)	152
N2—H2⋯O1*W* ^iv^	0.88	2.03	2.852 (4)	155
N3—H3⋯O1*W* ^iv^	0.88	2.18	2.968 (4)	149
O1*W*—H1*WA*⋯O4	0.92	1.90	2.786 (4)	162
O1*W*—H1*WB*⋯O2^v^	0.86	2.04	2.873 (4)	164
O2*W*—H2*WA*⋯O5	0.87	2.48	3.055 (18)	124
O2*W*—H2*WB*⋯O5^vi^	1.09	2.27	3.125 (18)	134

Symmetry codes: (i) 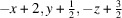 ; (iii) 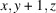 ; (iv) 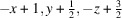 ; (v) 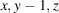 ; (vi) 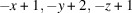 .

**Table 3:** Experimental details

Crystal data	
Chemical formula	[Cu(C_14_H_8_O_4_)(C_11_H_10_N_4_O)(H_2_O)]·1.25H_2_O
*M*_r_	558.51
Crystal system, space group	Monoclinic, *P*2_1_/*c*
Temperature (K)	173
*a*, *b*, *c* (Å)	10.3175 (8), 10.3927 (8), 22.9891 (18)
β (°)	100.133 (1)
*V* (Å^3^)	2426.6 (3)
*Z*	4
Radiation type	Mo *K*α
μ (mm^−1^)	0.96
Crystal size (mm)	0.21 × 0.18 × 0.08

Data collection	
Diffractometer	Bruker APEXII CCD
Absorption correction	Multi-scan (*SADABS*; Bruker, 2015 ▸)
*T*_min_, *T*_max_	0.657, 0.745
No. of measured, independent and observed [*I* > 2σ(*I*)] reflections	19136, 4459, 3228
*R*_int_	0.069
(sin θ/λ)_max_ (Å^−1^)	0.603

Refinement	
*R*[*F* ^2^ > 2σ(*F* ^2^)], *wR*(*F* ^2^), *S*	0.051, 0.133, 1.05
No. of reflections	4459
No. of parameters	344
No. of restraints	3
H-atom treatment	H-atom parameters constrained
Δρ_max_, Δρ_min_ (e Å^−3^)	0.55, −0.36

Computer programs: *COSMO* (Bruker, 2009 ▸), *SAINT* (Bruker, 2013 ▸), *SHELXT* (Sheldrick, 2015*a* ▸), *SHELXL* (Sheldrick, 2015*b* ▸) and *OLEX2* (Dolomanov *et al.*, 2009 ▸).

## full crystallographic data

### Crystal data

**Table:**

[Cu(C_14_H_8_O_4_)(C_11_H_10_N_4_O)(H_2_O)]·1.25H_2_O	*F*(000) = 1150
*M**_r_* = 558.51	*D*_x_ = 1.529 Mg m^−^^3^
Monoclinic, *P*2_1_/*c*	Mo *K*α radiation, λ = 0.71073 Å
*a* = 10.3175 (8) Å	Cell parameters from 4863 reflections
*b* = 10.3927 (8) Å	θ = 2.5–24.7°
*c* = 22.9891 (18) Å	µ = 0.96 mm^−^^1^
β = 100.133 (1)°	*T* = 173 K
*V* = 2426.6 (3) Å^3^	Block, green
*Z* = 4	0.21 × 0.18 × 0.08 mm

### Data collection

**Table:**

Bruker APEXII CCD diffractometer	4459 independent reflections
Radiation source: sealed tube	3228 reflections with *I* > 2σ(*I*)
Graphite monochromator	*R*_int_ = 0.069
Detector resolution: 8.36 pixels mm^-1^	θ_max_ = 25.4°, θ_min_ = 1.8°
ω scans	*h* = −12→12
Absorption correction: multi-scan (SADABS; Bruker, 2015)	*k* = −12→12
*T*_min_ = 0.657, *T*_max_ = 0.745	*l* = −27→27
19136 measured reflections	

### Refinement

**Table:**

Refinement on *F*^2^	Primary atom site location: dual
Least-squares matrix: full	Hydrogen site location: mixed
*R*[*F*^2^ > 2σ(*F*^2^)] = 0.051	H-atom parameters constrained
*wR*(*F*^2^) = 0.133	*w* = 1/[σ^2^(*F*_o_^2^) + (0.0655*P*)^2^ + 0.2521*P*] where *P* = (*F*_o_^2^ + 2*F*_c_^2^)/3
*S* = 1.05	(Δ/σ)_max_ < 0.001
4459 reflections	Δρ_max_ = 0.55 e Å^−^^3^
344 parameters	Δρ_min_ = −0.35 e Å^−^^3^
3 restraints	

### Special details

**Table:**

Experimental. Data was collected using a BRUKER CCD (charge coupled device) based diffractometer equipped with an Oxford low-temperature apparatus operating at 173 K. A suitable crystal was chosen and mounted on a nylon loop using Paratone oil. Data were measured using omega scans of 0.5° per frame for 30 s. The total number of images were based on results from the program COSMO where redundancy was expected to be 4 and completeness to 0.83Å to 100%. Cell parameters were retrieved using APEX II software and refined using SAINT on all observed reflections.Data reduction was performed using the SAINT software which corrects for Lp. Scaling and absorption corrections were applied using SADABS6 multi-scan technique, supplied by George Sheldrick. The structure was solved by the direct method using the SHELXT program and refined by least squares method on F2, SHELXL, incorporated in OLEX2.
Geometry. All esds (except the esd in the dihedral angle between two l.s. planes) are estimated using the full covariance matrix. The cell esds are taken into account individually in the estimation of esds in distances, angles and torsion angles; correlations between esds in cell parameters are only used when they are defined by crystal symmetry. An approximate (isotropic) treatment of cell esds is used for estimating esds involving l.s. planes.
Refinement. The structure was refined by Least Squares using version 2018/3 of XL (Sheldrick, 2015) incorporated in Olex2 (Dolomanov *et al.*, 2009). All non-hydrogen atoms were refined anisotropically. Hydrogen atom positions were calculated geometrically and refined using the riding model, except for the Hydrogen atom on the nitrogen atom which was found by difference Fourier methods and refined isotropically.

### Fractional atomic coordinates and isotropic or equivalent isotropic displacement parameters (Å^2^)

**Table:**

	*x*	*y*	*z*	*U*_iso_*/*U*_eq_	Occ. (<1)
Cu1	0.95431 (4)	0.76599 (4)	0.77058 (2)	0.02581 (16)	
O1	0.9668 (2)	0.6029 (2)	0.81409 (10)	0.0297 (6)	
O2	0.8465 (3)	0.6933 (2)	0.87424 (12)	0.0416 (7)	
O3	0.8956 (3)	0.2538 (2)	0.78666 (13)	0.0392 (7)	
O4	0.7695 (3)	0.0878 (2)	0.80223 (13)	0.0445 (8)	
O5	0.5035 (3)	0.7162 (3)	0.50224 (13)	0.0508 (8)	
O6	0.8988 (2)	0.9403 (2)	0.74133 (11)	0.0311 (6)	
H6A	0.9660	0.9824	0.7308	0.047*	
H6B	0.8785	0.9878	0.7705	0.047*	
N1	0.8120 (3)	0.6945 (3)	0.70842 (13)	0.0270 (7)	
N2	0.4890 (3)	0.5856 (3)	0.58155 (13)	0.0322 (8)	
H2	0.4415	0.5233	0.5931	0.039*	
N3	0.3297 (3)	0.5784 (3)	0.49940 (13)	0.0321 (7)	
H3	0.2996	0.5154	0.5189	0.039*	
N4	0.0890 (3)	0.6655 (3)	0.33768 (13)	0.0292 (7)	
C1	0.9148 (4)	0.6047 (3)	0.86083 (16)	0.0278 (8)	
C2	0.9467 (4)	0.4880 (3)	0.90033 (15)	0.0258 (8)	
C3	1.0709 (4)	0.4818 (4)	0.93635 (16)	0.0324 (9)	
H3A	1.1325	0.5491	0.9349	0.039*	
C4	1.1048 (4)	0.3788 (4)	0.97396 (16)	0.0351 (9)	
H4	1.1887	0.3761	0.9988	0.042*	
C5	1.0159 (4)	0.2797 (4)	0.97530 (17)	0.0357 (10)	
H5	1.0383	0.2093	1.0015	0.043*	
C6	0.8942 (4)	0.2830 (4)	0.93849 (17)	0.0363 (10)	
H6	0.8348	0.2133	0.9387	0.044*	
C7	0.8574 (4)	0.3885 (3)	0.90069 (16)	0.0288 (9)	
C8	0.7259 (4)	0.3897 (3)	0.86209 (17)	0.0316 (9)	
C9	0.6290 (4)	0.4775 (4)	0.87134 (19)	0.0407 (10)	
H9	0.6473	0.5379	0.9027	0.049*	
C10	0.5066 (4)	0.4771 (4)	0.8353 (2)	0.0492 (12)	
H10	0.4423	0.5383	0.8420	0.059*	
C11	0.4763 (4)	0.3902 (4)	0.7901 (2)	0.0433 (11)	
H11	0.3925	0.3919	0.7651	0.052*	
C12	0.5699 (4)	0.2998 (4)	0.78130 (19)	0.0377 (10)	
H12	0.5484	0.2368	0.7512	0.045*	
C13	0.6941 (4)	0.3000 (3)	0.81582 (17)	0.0299 (9)	
C14	0.7959 (4)	0.2074 (4)	0.80076 (16)	0.0295 (9)	
C15	0.7264 (4)	0.6020 (3)	0.71812 (17)	0.0307 (9)	
H15	0.7399	0.5601	0.7554	0.037*	
C16	0.6215 (4)	0.5662 (3)	0.67659 (16)	0.0322 (9)	
H16	0.5631	0.5014	0.6855	0.039*	
C17	0.6000 (3)	0.6242 (3)	0.62140 (16)	0.0282 (8)	
C18	0.6892 (4)	0.7167 (3)	0.61037 (16)	0.0303 (9)	
H18	0.6794	0.7577	0.5729	0.036*	
C19	0.7921 (4)	0.7478 (3)	0.65451 (17)	0.0309 (9)	
H19	0.8530	0.8111	0.6463	0.037*	
C20	0.4464 (4)	0.6351 (4)	0.52610 (16)	0.0329 (9)	
C21	0.0631 (4)	0.5687 (4)	0.37300 (17)	0.0321 (9)	
H21	−0.0137	0.5184	0.3607	0.039*	
C22	0.1413 (4)	0.5392 (3)	0.42540 (17)	0.0327 (9)	
H22	0.1184	0.4701	0.4486	0.039*	
C23	0.2551 (4)	0.6106 (3)	0.44496 (16)	0.0277 (8)	
C24	0.2860 (4)	0.7074 (4)	0.40816 (17)	0.0331 (9)	
H24	0.3642	0.7565	0.4188	0.040*	
C25	0.2010 (4)	0.7312 (3)	0.35587 (17)	0.0309 (9)	
H25	0.2229	0.7982	0.3312	0.037*	
O1W	0.7281 (3)	−0.0656 (3)	0.89666 (14)	0.0534 (8)	
H1WA	0.7247	−0.0227	0.8615	0.080*	
H1WB	0.7482	−0.1419	0.8873	0.080*	
O2W	0.4163 (16)	0.9950 (17)	0.4781 (9)	0.109 (6)	0.25
H2WA	0.4441	0.9313	0.4591	0.164*	0.25
H2WB	0.4946	1.0663	0.4874	0.164*	0.25

### Atomic displacement parameters (Å^2^)

**Table:**

	*U*^11^	*U*^22^	*U*^33^	*U*^12^	*U*^13^	*U*^23^
Cu1	0.0267 (3)	0.0211 (3)	0.0296 (3)	−0.00063 (19)	0.00458 (19)	0.00122 (18)
O1	0.0414 (16)	0.0211 (13)	0.0276 (15)	−0.0011 (11)	0.0090 (12)	0.0012 (10)
O2	0.0485 (18)	0.0313 (16)	0.0497 (18)	0.0106 (14)	0.0221 (15)	0.0067 (13)
O3	0.0289 (15)	0.0466 (18)	0.0440 (18)	−0.0064 (13)	0.0115 (13)	−0.0084 (13)
O4	0.0420 (17)	0.0271 (16)	0.067 (2)	0.0036 (13)	0.0167 (15)	−0.0046 (14)
O5	0.0465 (18)	0.061 (2)	0.0392 (18)	−0.0210 (16)	−0.0078 (15)	0.0174 (15)
O6	0.0362 (16)	0.0199 (13)	0.0376 (16)	0.0017 (11)	0.0081 (12)	0.0021 (11)
N1	0.0301 (17)	0.0224 (16)	0.0289 (18)	−0.0027 (13)	0.0061 (14)	0.0018 (13)
N2	0.0296 (18)	0.0345 (18)	0.0323 (19)	−0.0067 (14)	0.0049 (14)	0.0051 (14)
N3	0.0307 (18)	0.0334 (18)	0.0321 (19)	−0.0042 (14)	0.0047 (15)	0.0020 (14)
N4	0.0270 (17)	0.0227 (16)	0.0373 (19)	0.0013 (13)	0.0039 (14)	−0.0009 (14)
C1	0.030 (2)	0.0214 (19)	0.031 (2)	−0.0023 (16)	0.0029 (17)	−0.0036 (16)
C2	0.034 (2)	0.0225 (19)	0.0233 (19)	0.0034 (16)	0.0102 (17)	−0.0019 (15)
C3	0.044 (2)	0.027 (2)	0.027 (2)	0.0011 (18)	0.0090 (19)	−0.0012 (16)
C4	0.039 (2)	0.035 (2)	0.030 (2)	0.0130 (19)	0.0023 (18)	−0.0029 (17)
C5	0.052 (3)	0.029 (2)	0.027 (2)	0.0075 (19)	0.010 (2)	0.0011 (16)
C6	0.050 (3)	0.027 (2)	0.035 (2)	−0.0014 (18)	0.018 (2)	0.0031 (17)
C7	0.036 (2)	0.026 (2)	0.027 (2)	0.0004 (16)	0.0138 (17)	−0.0024 (16)
C8	0.038 (2)	0.0210 (19)	0.039 (2)	−0.0005 (16)	0.0165 (19)	0.0046 (16)
C9	0.042 (3)	0.033 (2)	0.049 (3)	0.0040 (19)	0.015 (2)	−0.0054 (19)
C10	0.038 (3)	0.037 (3)	0.077 (4)	0.012 (2)	0.022 (2)	0.004 (2)
C11	0.027 (2)	0.043 (3)	0.060 (3)	0.0014 (19)	0.009 (2)	0.014 (2)
C12	0.035 (2)	0.033 (2)	0.047 (3)	−0.0069 (18)	0.012 (2)	0.0043 (19)
C13	0.024 (2)	0.027 (2)	0.040 (2)	−0.0026 (16)	0.0106 (18)	0.0074 (17)
C14	0.025 (2)	0.038 (2)	0.025 (2)	−0.0004 (17)	0.0040 (17)	−0.0014 (16)
C15	0.032 (2)	0.030 (2)	0.030 (2)	−0.0004 (17)	0.0060 (17)	0.0052 (16)
C16	0.034 (2)	0.027 (2)	0.036 (2)	−0.0066 (17)	0.0072 (18)	0.0008 (17)
C17	0.027 (2)	0.027 (2)	0.031 (2)	0.0037 (16)	0.0047 (17)	−0.0024 (16)
C18	0.035 (2)	0.033 (2)	0.023 (2)	−0.0035 (17)	0.0057 (17)	0.0011 (16)
C19	0.031 (2)	0.028 (2)	0.035 (2)	−0.0042 (16)	0.0078 (17)	0.0012 (17)
C20	0.035 (2)	0.034 (2)	0.030 (2)	−0.0003 (18)	0.0056 (18)	−0.0010 (17)
C21	0.029 (2)	0.032 (2)	0.035 (2)	−0.0023 (17)	0.0049 (18)	0.0002 (17)
C22	0.035 (2)	0.024 (2)	0.039 (2)	−0.0040 (17)	0.0042 (18)	0.0043 (17)
C23	0.030 (2)	0.025 (2)	0.029 (2)	0.0029 (16)	0.0060 (17)	−0.0010 (16)
C24	0.029 (2)	0.031 (2)	0.039 (2)	−0.0047 (17)	0.0036 (18)	−0.0016 (17)
C25	0.031 (2)	0.027 (2)	0.034 (2)	0.0012 (17)	0.0032 (18)	0.0019 (16)
O1W	0.060 (2)	0.0444 (18)	0.061 (2)	0.0091 (15)	0.0258 (17)	−0.0006 (15)
O2W	0.083 (12)	0.093 (14)	0.152 (18)	0.000 (11)	0.022 (12)	−0.007 (12)

### Geometric parameters (Å, º)

**Table:**

Cu1—O1	1.960 (2)	C6—C7	1.409 (5)
Cu1—O3^i^	2.205 (3)	C7—C8	1.485 (5)
Cu1—O6	1.981 (2)	C8—C9	1.397 (5)
Cu1—N1	2.003 (3)	C8—C13	1.409 (5)
Cu1—N4^ii^	2.016 (3)	C9—H9	0.9500
O1—C1	1.283 (4)	C9—C10	1.383 (6)
O2—C1	1.231 (4)	C10—H10	0.9500
O3—Cu1^iii^	2.205 (3)	C10—C11	1.372 (6)
O3—C14	1.230 (4)	C11—H11	0.9500
O4—C14	1.274 (4)	C11—C12	1.387 (6)
O5—C20	1.213 (4)	C12—H12	0.9500
O6—H6A	0.8881	C12—C13	1.383 (5)
O6—H6B	0.8878	C13—C14	1.510 (5)
N1—C15	1.351 (4)	C15—H15	0.9500
N1—C19	1.340 (5)	C15—C16	1.363 (5)
N2—H2	0.8800	C16—H16	0.9500
N2—C17	1.393 (5)	C16—C17	1.387 (5)
N2—C20	1.373 (5)	C17—C18	1.385 (5)
N3—H3	0.8800	C18—H18	0.9500
N3—C20	1.384 (5)	C18—C19	1.372 (5)
N3—C23	1.390 (5)	C19—H19	0.9500
N4—Cu1^iv^	2.016 (3)	C21—H21	0.9500
N4—C21	1.349 (5)	C21—C22	1.362 (5)
N4—C25	1.344 (5)	C22—H22	0.9500
C1—C2	1.516 (5)	C22—C23	1.394 (5)
C2—C3	1.399 (5)	C23—C24	1.387 (5)
C2—C7	1.386 (5)	C24—H24	0.9500
C3—H3A	0.9500	C24—C25	1.380 (5)
C3—C4	1.382 (5)	C25—H25	0.9500
C4—H4	0.9500	O1W—H1WA	0.9177
C4—C5	1.383 (6)	O1W—H1WB	0.8564
C5—H5	0.9500	O2W—H2WA	0.8696
C5—C6	1.386 (6)	O2W—H2WB	1.0893
C6—H6	0.9500		
			
O1—Cu1—O3^i^	105.34 (10)	C10—C9—C8	120.7 (4)
O1—Cu1—O6	162.28 (11)	C10—C9—H9	119.7
O1—Cu1—N1	91.02 (11)	C9—C10—H10	119.4
O1—Cu1—N4^ii^	86.57 (11)	C11—C10—C9	121.2 (4)
O6—Cu1—O3^i^	92.38 (10)	C11—C10—H10	119.4
O6—Cu1—N1	88.00 (11)	C10—C11—H11	120.5
O6—Cu1—N4^ii^	93.20 (11)	C10—C11—C12	119.0 (4)
N1—Cu1—O3^i^	93.24 (12)	C12—C11—H11	120.5
N1—Cu1—N4^ii^	175.68 (12)	C11—C12—H12	119.6
N4^ii^—Cu1—O3^i^	90.86 (12)	C13—C12—C11	120.9 (4)
C1—O1—Cu1	114.5 (2)	C13—C12—H12	119.6
C14—O3—Cu1^iii^	152.6 (3)	C8—C13—C14	121.1 (3)
Cu1—O6—H6A	110.5	C12—C13—C8	120.2 (4)
Cu1—O6—H6B	110.1	C12—C13—C14	118.6 (3)
H6A—O6—H6B	103.3	O3—C14—O4	125.6 (4)
C15—N1—Cu1	124.4 (2)	O3—C14—C13	117.3 (3)
C19—N1—Cu1	118.7 (2)	O4—C14—C13	117.1 (3)
C19—N1—C15	116.7 (3)	N1—C15—H15	118.7
C17—N2—H2	116.8	N1—C15—C16	122.7 (3)
C20—N2—H2	116.8	C16—C15—H15	118.7
C20—N2—C17	126.4 (3)	C15—C16—H16	119.9
C20—N3—H3	116.6	C15—C16—C17	120.1 (3)
C20—N3—C23	126.7 (3)	C17—C16—H16	119.9
C23—N3—H3	116.6	C16—C17—N2	117.2 (3)
C21—N4—Cu1^iv^	122.8 (2)	C18—C17—N2	125.1 (3)
C25—N4—Cu1^iv^	119.8 (2)	C18—C17—C16	117.7 (3)
C25—N4—C21	116.3 (3)	C17—C18—H18	120.6
O1—C1—C2	114.3 (3)	C19—C18—C17	118.8 (3)
O2—C1—O1	124.2 (3)	C19—C18—H18	120.6
O2—C1—C2	121.5 (3)	N1—C19—C18	124.0 (3)
C3—C2—C1	118.1 (3)	N1—C19—H19	118.0
C7—C2—C1	121.7 (3)	C18—C19—H19	118.0
C7—C2—C3	120.2 (3)	O5—C20—N2	125.3 (4)
C2—C3—H3A	119.6	O5—C20—N3	123.3 (4)
C4—C3—C2	120.7 (4)	N2—C20—N3	111.3 (3)
C4—C3—H3A	119.6	N4—C21—H21	118.3
C3—C4—H4	120.2	N4—C21—C22	123.4 (4)
C3—C4—C5	119.7 (4)	C22—C21—H21	118.3
C5—C4—H4	120.2	C21—C22—H22	120.0
C4—C5—H5	120.0	C21—C22—C23	120.0 (4)
C4—C5—C6	120.1 (4)	C23—C22—H22	120.0
C6—C5—H5	120.0	N3—C23—C22	117.6 (3)
C5—C6—H6	119.6	C24—C23—N3	125.0 (3)
C5—C6—C7	120.9 (4)	C24—C23—C22	117.4 (3)
C7—C6—H6	119.6	C23—C24—H24	120.6
C2—C7—C6	118.5 (4)	C25—C24—C23	118.8 (4)
C2—C7—C8	121.9 (3)	C25—C24—H24	120.6
C6—C7—C8	119.6 (3)	N4—C25—C24	124.0 (4)
C9—C8—C7	121.2 (3)	N4—C25—H25	118.0
C9—C8—C13	118.0 (4)	C24—C25—H25	118.0
C13—C8—C7	120.9 (3)	H1WA—O1W—H1WB	101.6
C8—C9—H9	119.7	H2WA—O2W—H2WB	108.5
			
Cu1—O1—C1—O2	9.6 (5)	C7—C8—C13—C14	−5.2 (5)
Cu1—O1—C1—C2	−169.0 (2)	C8—C9—C10—C11	0.8 (6)
Cu1^iii^—O3—C14—O4	44.2 (8)	C8—C13—C14—O3	−59.1 (5)
Cu1^iii^—O3—C14—C13	−133.8 (5)	C8—C13—C14—O4	122.8 (4)
Cu1—N1—C15—C16	172.2 (3)	C9—C8—C13—C12	−0.4 (5)
Cu1—N1—C19—C18	−172.8 (3)	C9—C8—C13—C14	176.3 (3)
Cu1^iv^—N4—C21—C22	−165.6 (3)	C9—C10—C11—C12	1.2 (6)
Cu1^iv^—N4—C25—C24	166.3 (3)	C10—C11—C12—C13	−2.8 (6)
O1—C1—C2—C3	77.6 (4)	C11—C12—C13—C8	2.4 (6)
O1—C1—C2—C7	−101.5 (4)	C11—C12—C13—C14	−174.4 (4)
O2—C1—C2—C3	−101.1 (4)	C12—C13—C14—O3	117.7 (4)
O2—C1—C2—C7	79.8 (5)	C12—C13—C14—O4	−60.4 (5)
N1—C15—C16—C17	0.8 (6)	C13—C8—C9—C10	−1.1 (6)
N2—C17—C18—C19	178.1 (3)	C15—N1—C19—C18	2.1 (5)
N3—C23—C24—C25	−178.3 (3)	C15—C16—C17—N2	−178.3 (3)
N4—C21—C22—C23	−0.2 (6)	C15—C16—C17—C18	1.0 (5)
C1—C2—C3—C4	179.0 (3)	C16—C17—C18—C19	−1.2 (5)
C1—C2—C7—C6	179.9 (3)	C17—N2—C20—O5	4.0 (6)
C1—C2—C7—C8	0.6 (5)	C17—N2—C20—N3	−177.4 (3)
C2—C3—C4—C5	1.1 (5)	C17—C18—C19—N1	−0.3 (6)
C2—C7—C8—C9	−68.6 (5)	C19—N1—C15—C16	−2.4 (5)
C2—C7—C8—C13	113.0 (4)	C20—N2—C17—C16	176.6 (4)
C3—C2—C7—C6	0.8 (5)	C20—N2—C17—C18	−2.7 (6)
C3—C2—C7—C8	−178.5 (3)	C20—N3—C23—C22	178.6 (3)
C3—C4—C5—C6	0.9 (6)	C20—N3—C23—C24	−0.5 (6)
C4—C5—C6—C7	−2.1 (6)	C21—N4—C25—C24	−1.9 (5)
C5—C6—C7—C2	1.2 (5)	C21—C22—C23—N3	178.5 (3)
C5—C6—C7—C8	−179.5 (3)	C21—C22—C23—C24	−2.2 (5)
C6—C7—C8—C9	112.2 (4)	C22—C23—C24—C25	2.5 (5)
C6—C7—C8—C13	−66.3 (5)	C23—N3—C20—O5	−5.5 (6)
C7—C2—C3—C4	−1.9 (5)	C23—N3—C20—N2	175.8 (3)
C7—C8—C9—C10	−179.6 (4)	C23—C24—C25—N4	−0.5 (6)
C7—C8—C13—C12	178.1 (3)	C25—N4—C21—C22	2.2 (5)

Symmetry codes: (i) −*x*+2, *y*+1/2, −*z*+3/2; (ii) *x*+1, −*y*+3/2, *z*+1/2; (iii) −*x*+2, *y*−1/2, −*z*+3/2; (iv) *x*−1, −*y*+3/2, *z*−1/2.

### Hydrogen-bond geometry (Å, º)

**Table:**

*D*—H···*A*	*D*—H	H···*A*	*D*···*A*	*D*—H···*A*
O6—H6*A*···O1^i^	0.89	1.84	2.650 (3)	152
O6—H6*B*···O4^v^	0.89	1.78	2.598 (4)	152
N2—H2···O1*W*^vi^	0.88	2.03	2.852 (4)	155
N3—H3···O1*W*^vi^	0.88	2.18	2.968 (4)	149
O1*W*—H1*WA*···O4	0.92	1.90	2.786 (4)	162
O1*W*—H1*WB*···O2^vii^	0.86	2.04	2.873 (4)	164
O2*W*—H2*WA*···O5	0.87	2.48	3.055 (18)	124
O2*W*—H2*WB*···O5^viii^	1.09	2.27	3.125 (18)	134

Symmetry codes: (i) −*x*+2, *y*+1/2, −*z*+3/2; (v) *x*, *y*+1, *z*; (vi) −*x*+1, *y*+1/2, −*z*+3/2; (vii) *x*, *y*−1, *z*; (viii) −*x*+1, −*y*+2, −*z*+1.
